# Advancements in ultrafast photonics: confluence of nonlinear optics and intelligent strategies

**DOI:** 10.1038/s41377-024-01732-7

**Published:** 2025-02-25

**Authors:** Qing Wu, Liuxing Peng, Zhihao Huang, Xiaolei Liu, Meng Luo, Danheng Gao, Haoran Meng

**Affiliations:** 1 Heilongjiang Province Key Laboratory of Laser Spectroscopy Technology and Application, Harbin University of Science and Technology, Harbin, 150080 China; 2 State Key Laboratory of Applied Optics, Changchun Institute of Optics, Fine Mechanics and Physics, Chinese Academy of Sciences, Changchun, 130033 China

**Keywords:** Ultrafast lasers, Mode-locked lasers

## Abstract

Automatic mode-locking techniques, the integration of intelligent technologies with nonlinear optics offers the promise of on-demand intelligent control, potentially overcoming the inherent limitations of traditional ultrafast pulse generation that have predominantly suffered from the instability and suboptimality of open-loop manual tuning. The advancements in intelligent algorithm-driven automatic mode-locking techniques primarily are explored in this review, which also revisits the fundamental principles of nonlinear optical absorption, and examines the evolution and categorization of conventional mode-locking techniques. The convergence of ultrafast pulse nonlinear interactions with intelligent technologies has intricately expanded the scope of ultrafast photonics, unveiling considerable potential for innovation and catalyzing new waves of research breakthroughs in ultrafast photonics and nonlinear optics characters.

## Abstract (web summary)

Automatic mode-locking techniques, the integration of intelligent technologies with nonlinear optics offers the promise of on-demand intelligent control, potentially overcoming the inherent limitations of traditional ultrafast pulse systems.

## Graphical abstract

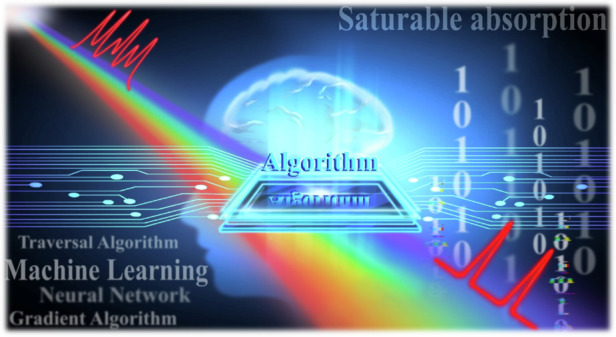

## Introduction

Ultrafast pulses have widespread applications, encompassing optical frequency metrology^1–3^, precision ranging^4–6^, precision manufacturing^7–10^, and astronomical observation^11,12^. Distinguished from conventional solid-state lasers, fiber lasers, which are characterized by their flexible waveguides, provide notable advantages^13^ including the elimination of collimation requirements, superior beam quality, and cost efficiency, thereby establishing themselves as a primary focus of research in the field of ultrafast lasers^14^ while concurrently offering an ideal platform for the exploration of nonlinear effects^15,16^. The generation of a picosecond (ps) or femtosecond-scale (fs) ultrafast pulses relies predominantly on Q-switching (QS)^17,18^ and mode-locking methodologies^19–21^. However, QS generally produces pulse durations on the nanosecond scale, thereby necessitating mode-locking techniques to achieve pulse widths at least an order of magnitude narrower than those generated by QS^22^. Fiber-based mode-locking techniques, which consist of active mode-locking, passive mode-locking (PML), and hybrid mode-locking, have been widely investigated for ultrafast pulse generation. Unlike active and hybrid mode-locking that necessitate the introduction of modulating devices within the resonator, leading to increased complexity and limited applicability, PML has become the most extensively applied and thoroughly researched method^23^ due to the inherent advantages of its compact structure, easy-to-implement, and capability of generating femtosecond pulses^24^.

In the pursuit of enhancing pulse energy, output power, etc., researchers have continuously explored novel PML mechanisms and advanced laser materials, leading to the constant introduction of various mode-locking techniques. Current research trends predominantly explore saturable absorbers based on nanomaterials^25,26^, nonlinear optical loop mirrors (NOLMs)^27,28^, nonlinear multimode interference (NLMMI)^29,30^, and nonlinear polarization evolution/rotation (NPE/NPR)^31–33^, Mamyshev oscillators, etc. Among these, the PML of fiber lasers based on NPR which exploits polarization control and Kerr nonlinearity^34^, has garnered attention due to structural simplicity^24,33,35^ and rich dynamical states^36–38^. Leveraging self-phase modulation and cross-phase modulation processes within the fiber^39^, pulses accrue varying degrees of nonlinear phase shifts across different intensity segments, refining the polarization state within the cavity. Consequently, as the signal traverses the polarizer, regions of high-intensity experience comparatively lower losses while regions of low intensity experience relatively high losses, effectuating efficient saturable absorption (SA)^34,40^, enabling the stable narrowing of pulse widths, aiding in the production of ultrafast pulses with high repetition-rates^41^ and swift response times. The complex interplay between nonlinearity, dispersion, gain, and loss within the resonant cavity produces abundant mode-locked states (ML), including fundamental frequency mode-locking (FML)^42,43^, high repetition-rate harmonic mode-locking (HML)^44,45^ and hybrid Q-switched mode-locking (QML)^46,47^. Therefore, the precise control of laser parameters to access specific mode-locking states remains a significant and challenging task^23,48^.

**Fig. 1:**
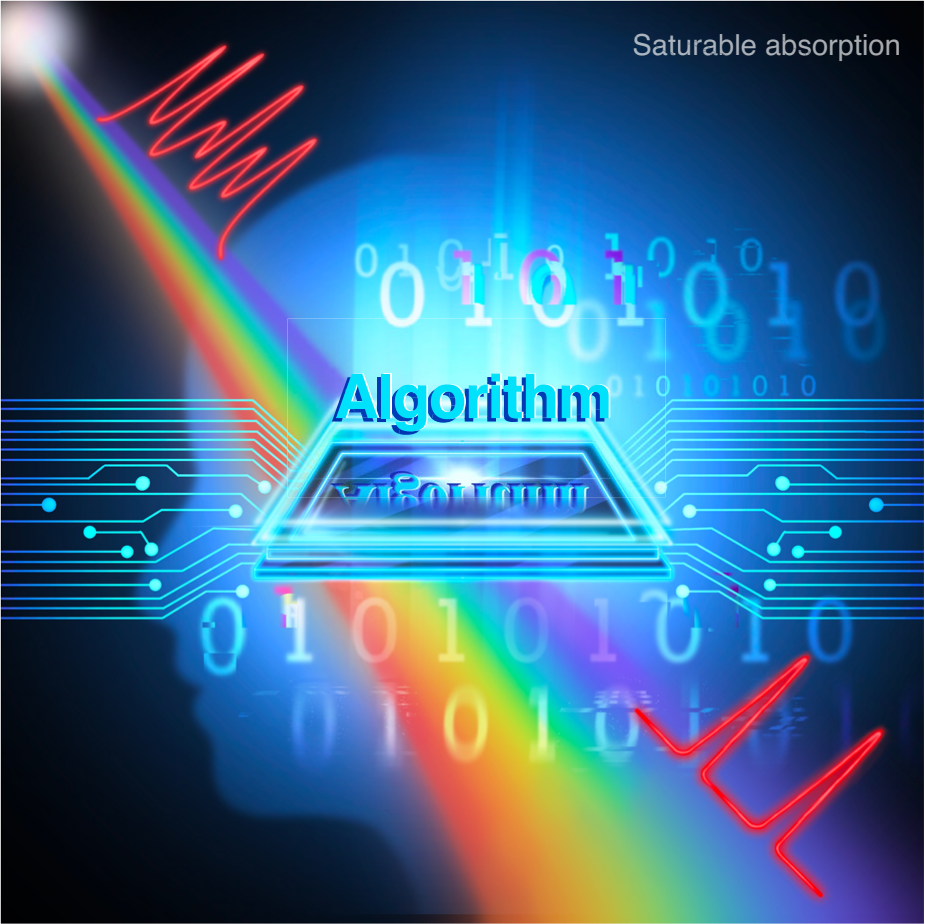
**The conceptual framework of AML technology**

Traditionally ultrafast pulses have been predominantly reliant on nonlinear optical effects and have typically combined open-loop manual tuning, which is incapable of achieving stable and optimal control. Recent research has introduced automatic mode-locking (AML) techniques that address the challenges posed by the complex landscape of mode-locking mechanisms, such as the difficulty in manually achieving the desired pulse modes and the constrained polarization state space for various pulse configurations, by precisely controlling the mode-locked states^49,50^. The conceptual framework of AML technology as illustrated in Fig. 1, emphasizes the pivotal roles of typically the closed-loop feedback system and intelligent control algorithms in this technique. The closed-loop feedback system is the integration of data acquisition modules, computational modules, and control modules dedicated to capturing output data, processing and analyzing data, and subsequently dictating the precise adjustments of the control module to the laser state^51–54^. Employed widely-used real-time computational chips such as Microcontroller Units (MCU), Central Process Units, field-programmable gate arrays (FPGA), or devices composed of them, integrating with intelligent algorithms and electronic polarization controller (EPC) within the resonator to navigate the ML. The system automatically discerns the pulse states of the laser, and dynamically modulates and precisely governs the polarization state of the laser. External voltage modulation enables EPC to replace manual intervention in the mode-locked process. This approach allows algorithms to quickly identify and maintain the polarization states for stable pulse output by rapidly adjusting the polarization state of the laser. Upon acquiring polarization states with integrated hardware, the system employs various intelligent algorithms to analyze and identify information, enabling the rapid transition to ML^55^. The polarization control algorithms applied necessitate high speed and precision, robust search capabilities, and swift convergence to ascertain optimal solutions. Presently, these algorithms encompass traversal algorithms^56^, evolutionary algorithms (including genetic algorithm)^50,57–61^, human-like algorithm (HLA), deep learning algorithms integrating diverse models^50,57,58^, gradient algorithms^59^, and simulated annealing algorithms^60–62^. These algorithms accurately discriminate between pulse states, variously expedite the initial mode-locked process, and swiftly restore mode-locked states upon system reboot, overcoming environmental disturbances.

In this manuscript, we provide a detailed exposition of the principles of nonlinear optical properties, summarizing SA for implementing mode-locking techniques and the transition from SA to reverse saturable absorption (RSA), as researchers have found that RSA can significantly impact the stability of output pulses with increased pump power. Following an overview of SA, we explore the evolution and categorization of conventional mode-locking techniques, encompassing active mode-locking, PML, and hybrid mode-locking to establish a fundamental understanding of mode-locked device configurations and developmental paths. AML techniques become a significant thematic component within this review with the advancements in AML techniques succinctly presented. By enabling disparate algorithms and models, AML techniques overcome challenges faced by traditional mode-locking (response times, pulse width, etc.), offering researchers strategic insights for advancing AML technology. Additionally, the majority of the published work to date has been concentrated on the algorithmic development of AML techniques, nonlinear optical properties, or mode-locking techniques, primarily emphasizing performance parameters. It is noteworthy that our comprehensive review of these three areas of research progress provides a theoretical framework to guide researchers in future innovation and interdisciplinary studies.

## Nonlinear optical properties and mode-locking technologies

### Nonlinear optical properties

**Fig. 2:**
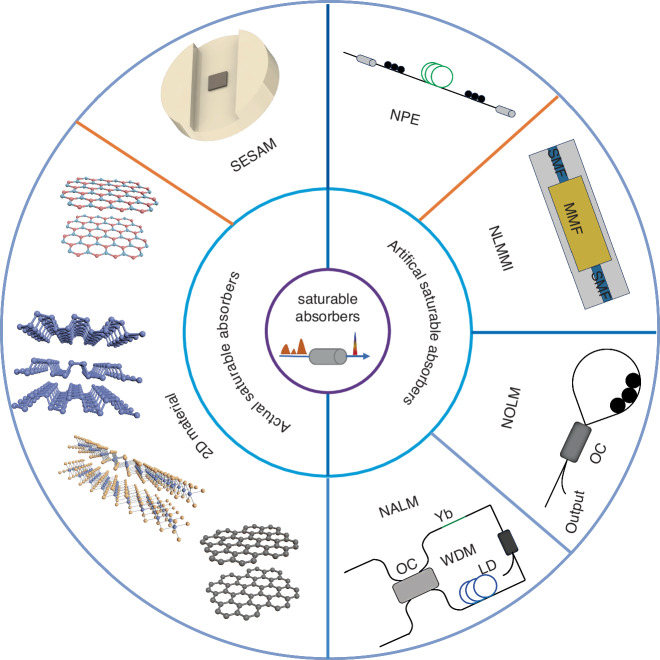
**Common saturable absorbers**

As a pivotal component for exploiting nonlinear effects to generate ultrafast pulses, saturable absorbers exploit their intensity-dependent characteristics to facilitate self-amplitude modulation of the input pulses within the cavity, necessitating low linear loss and high damage threshold to ensure the attainment of high-performance ultrafast fiber lasers. Depending on the method of realization, saturable absorbers can be categorized into actual saturable absorbers (ASA) and equivalent saturable absorbers (ESA)^63,64^, as presented in Fig. 2. ASA comprises semiconductor saturable absorber mirror (SESAM)^65,66^, nanomaterials such as carbon nanotubes (CNs)^67,68^, graphene^69–71^, and so on, which are cost-effective and straightforward to implement but are hampered by low damage thresholds and tendency of performance degradation over time. Alternatively, ESA induces varying degrees of nonlinear phase shift across different intensity segments of pulses, effectively constructing saturable absorbers, the pulses are continuously narrowed to generate ultrafast pulses^40^, including NLMMI^29,30^, NPE^31–33^, NOLM^27,28^, and Mamyshev regenerator. The parameters that characterize the performances of saturable absorbers include response time, saturation power, modulation depth, linear loss, and damage threshold^72–74^. The correlation between the response time and pulse width defines whether the absorber is a fast or slow saturable absorber. The level of saturation power directly impacts the mode-locking threshold of ultrafast pulses, with lower saturation power facilitating self-starting mode-locking^75^. With high power, slow saturable absorbers lead to pulse instability due to differential modulation of the leading and trailing edges of the pulse^76^, limiting the operating bandwidth of the system, which is detrimental to the generation of high-repetition rates. A large modulation depth is advantageous for the establishment and stabilization of mode-locking and can also narrow the pulse width^77^. This dynamic process involves the excitation of electrons (or holes) from low to high energy states following photon absorption, accompanied by a complex series of processes. This behavior is modeled through energy level transitions and is delineated into two categories: SA and RSA^78^. The SA utilizes the imaginary part of the third-order nonlinearity, I_m_(χ^3^), leveraging photon-induced electronic transitions and the Pauli exclusion principle to manifest as an intrinsic self-saturating phenomenon. In conditions where only ground-state nonlinear absorption is present, the medium exhibits SA with increasing light intensity. Conversely, RSA occurs when excited-state nonlinear absorption takes place, where molecules or atoms in a low-level excited state absorb photons and transition to the high excited state. RSA modulation, in which the ability to attenuate light intensity increases with increasing light intensity and influences the pulse output. RSA can cause the pulse to transition from dissipative soliton to dissipative soliton resonance, significantly affecting the quality of the mode-locked pulse^79^. The absorption cross-section of the excited state is larger than that of the ground state, material transmittance decreases under high intensity, which can be explained by energy level transition models for SA and RSA. Generally, when SA/RSA occurs, the absorption coefficient of the medium *α*(*I*) can be expressed as^80–82^:

1 $$a\left(I\right)=\frac{{a}_{0}}{1+\left(\frac{I}{{I}_{s}}\right)}+\beta I$$

where *α*_0_ is the linear absorption coefficient of the medium, *I*_*S*_ is the saturation light intensity, depending on the characterize of the medium, *β* expresses the nonlinear absorption coefficient. Upon excitation, photo-generated carriers transition to even higher excited states, where the absorption by these states exceeds that of the ground state, resulting in two-photon absorption (TPA) becoming the dominant mechanism in the nonlinear response and thereby leading to RSA^83,84^. Since RSA excites the excited electron from the low conduction band to the high conduction band by absorbing the second photon, this process is also called excited-state absorption (ESA)^85–87^ and typically occurs on ps and ns timescales.

**Fig. 3:**
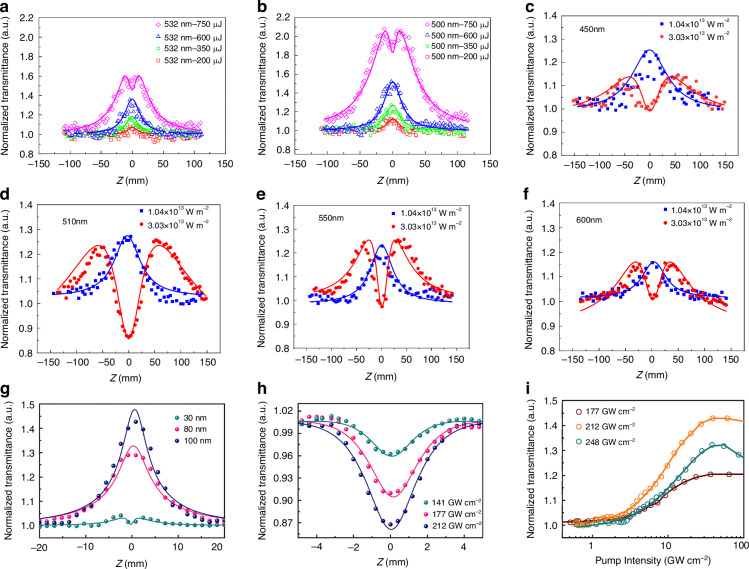
**Open aperture Z-scan data with different pump power**. **a**, **b** Normalized open aperture (OA) Z-scan transmittance of WS_2_ at different wavelengths (532 nm and 500 nm) across laser energies of 200 μJ, 350 μJ, 600 μJ, and 750 μJ. The dots are experimental data while the solid lines are theoretical data. Adapted with permission from ref. ^90^ ©Springer Nature. Normalized OA Z-scan transmittance of Au-Ag nanoparticles was obtained at **c** 450 nm, **d** 510 nm, **e** 550 nm, and **f** 600 nm. The dots are experimental data and the solid lines are theoretical data. Adapted with permission from ref. ^78^ ©MDPI. **g** Normalized transmittance of MoTe_2_ films with different thicknesses when the pumping intensity is 141 GW cm^−2^ (**h**) Normalized transmittance with different pump power of MoTe_2_ film (30 nm in thickness). **i** OA Z-scan curves of the 80 nm thick MoTe_2_ film at the different pump intensity. Adapted with permission from ref. ^85^ ©Elsevier

RSA typically manifests in non-resonant or near-resonant regions, enabling high linear transmission suitable for optical limiting^88^, whereas SA generally occurs within resonant regions. Significantly, in cases where the laser pulse duration approaches the lifetime of the highly excited state and the energy density of the laser is sufficiently high, the direct transition between SA and RSA can occur^89^. Researchers have employed a four-level model to analyze the competition between ground state and excited state absorption in various 2D materials, deducing that the SA-to-RSA transition is influenced by pump intensity, the absorption cross-section, and inter-level transition probabilities, investigating the characteristics of nonlinear optical responses^90^. Under identical pump energy, as depicted in Fig. 3g, h, thicker films (100 nm) exhibit SA, whereas thinner films (30 nm) display RSA^85^. Moreover, even for consistent film thickness, the transition from SA to RSA occurs with increasing pump power as shown in Fig. 3i^85^. MoSe_2_ nanosheets under 1 μm wavelength laser pulses manifest pronounced nonlinear SA and RSA^91^, transitioning from SA to RSA with increased pump power, attributed to the interplay between single-photon and TPA processes within the material. Employing a four-level model to analyze the transition between ground-state and ESA, the transition from SA to RSA is derived as being influenced by pump intensity, absorption cross-section, and transition probabilities between energy levels.

Researchers revealed that WS_2_ nanosheets demonstrate a notable SA at low excitation intensities. The effect is more pronounced at 500 nm compared with 532 nm as shown in Fig. 3a, b^90^. Moreover, Wang et al. researched Au-Ag nanoparticles (NPs), observing that the NPs steadily approach the focal point under a pump power of 760 μJ. The normalized transmittance initially increased and then decreased, indicating a transition from SA to RSA, as delineated in Fig. 3c–f^78^. Zheng et al. explored the nonlinear response of black phosphorus nanosheets using a Z-scan with an 800 nm femtosecond pulsed laser^92^. The nonlinear absorption was seen to transition from SA to RSA with escalating laser intensity. Mushtaq et al. reported on the nonlinear optical properties of layered aniline lead-brominated perovskite microdisks^93^, where optical nonlinearity shifted from SA to RSA with increased laser intensity. This nonlinearity is attributed to the interplay between single-photon and TPA processes in the material.

In the context of employing 2D materials as saturable absorbers, the emergence of RSA proves undesirable, considering that ML depends on the accumulation of high-intensity light within the cavity, where RSA inhibits the transmission of high-intensity light, resulting in the attenuation of the high-intensity components of pulses. The attenuation leads to pulse broadening, which contradicts the objective of achieving ultrafast pulses, impeding the initiation of the mode-locking mechanism, making it difficult to enter ML, increasing pulse width, and lowering output power.

Beyond affecting the performance of 2D materials as saturable absorbers, the presence of RSA also impacts the nonlinear effects in other mode-locking techniques. Excessive absorption of high-intensity light induced by RSA within the cavity results in increased transmission losses, significantly weakening the nonlinear effects, which directly impairs the formation of mode-locking and, in some cases, renders it unattainable. NPR relies on the variation of polarization states within the cavity. Nonlinear loop mirror (NLM) techniques achieve ML through nonlinear phase shifts in the loop cavity, but RSA increases the loss within the loop cavity, resulting in insufficient light intensity to produce the necessary phase shifts. Future research could further explore the impact of nonlinear SA transitions on mode-locked pulses in various mode-locking techniques to improve the performance of systems.

### Mode-locking technologies

#### Passive mode-locking

PML technology is the predominant approach for generating ultrafast pulses due to its intrinsic advantages^94,95^. It only uses the nonlinear effect of the gain material itself or saturable absorbers, enabling the generation of ultrafast pulses without additional modulators^96,97^. Saturable absorbers serve as a critical component in passive mode-locking fiber laser (PMLFLs), tasking with pulse narrowing and shaping to achieve ML, the self-amplitude modulation of pulses can be generated by the intensity dependence of the SA on the input pulse. When pulses hit the SA, the nonlinear absorption effect attenuates lower-energy pulses while allowing high-energy pulses to pass with minimal loss. After the iterative procedure, pulse narrowing occurs with concurrent phase fixation, repeating circulation within the cavity leads to progressive attenuation of both leading and trailing edges, culminating in laser stabilization^98^. Different PML techniques have been developed, including NLM^27,28,99^, NPR, and NLMMI^29,30^, etc.

Nonlinear amplifying loop mirrors (NALM) and NOLMs, fundamental Sagnac interferometers extensively utilized in mode-locking fiber laser (MLFL), exploiting the principles of interference inherent to NLM technologies to exhibit exceptional environmental robustness. All-fiber laser based on NOLM structure with an average power of 2.06 W and single pulse energy of 684 nJ^100^ is reported while NALM structure ensures high-repetition rate of 112.4 MHz^101^, output power of 126 mW^102^ and pulse duration of 88.7 fs^103^. NLM technology requires long intracavity single-mode fibers (SMFs) to provide sufficient nonlinear phase shift and high repetition-rate pulse output. Moreover, the intricacies of the NLM present inherent hurdles to realize the self-starting and it is necessary to implement the mode-locked pulse by applying external stress induction. Additionally, NLM technology configurations, reliant on strong nonlinear effects within the ring cavity to sustain stable pulse output, suffer from additional energy losses in the nonlinear medium due to RSA, which reduces pulse intensity and consequently fails to generate the requisite nonlinear phase shift. Furthermore, the excessive absorption caused by RSA induces instability in pulse shape and width, leading to noise-like pulses (NLPs) or frequent disruptions in the mode-locked state.

MLFLs employ SESAMs to distinguish themselves by low threshold, straightforward architecture, exceptional self-starting capabilities, and proficiency in delivering direct high pulse energy outputs of 2.4 W^104^. The advantage of the proposed configuration is the fast tuning of the pulse repetition rate in the range from tens of MHz to 1 GHz^105^. The lasers are capable of achieving output pulses of 380 fs^106^, central wavelength of 2 μm, and pulse energy of 440 nJ^107^. However, the inherent limitations of response time and narrow reflection bandwidth present significant challenges in realizing a broad spectrum of femtosecond-level mode-locked pulses. Concurrently, to meet the requirements of saturation intensity of SESAM, the laser must be focused onto the SESAM via lens transition, which predisposes the equipment to be damaged under conditions of high-power density. Therefore, the quest for long-term operational stability remains a pivotal concern in advancing and applying. In SESAM-based systems, broad absorption bandwidth enables efficient mode-locking by preferential transmission of high-intensity pulses, whereas RSA reduces the absorption bandwidth and impairs the ability to transmit high-energy pulses, which compromises both the stability and the spectral range of the output pulses. This ultimately results in reduced pulse energy, insufficient pulse compression, and increased pulse width, precluding the generation of high-quality pulses.

Mamyshev mechanism, a novel entrant predicated on nonlinear spectral broadening mode-locked oscillators techniques introduced in recent years^108–110^, holds substantial applicative value for generating ultrafast pulses with excellent spectral bandwidth and pulse energy. Liu et al. used large mode-field photonic crystal fiber to build Mamyshev oscillator^111^, which realized pulsed output with pulse energy of 1.125 μJ, repetition rate of 8 MHz, and peak power of 13 mW. Advancements continued in 2021 when the Wise research group first proposed and realized the Mamyshev mechanism with an optical quasi-molecule structure, culminating in pulses with the energy of 21 nJ, repetition frequency of 16.8 MHz, and pulse duration of 65 fs^112^. Typically, the implementation of the Mamyshev mechanism within the cavity necessitates active modulation or the introduction of external seeds. One thing to note is that Mamyshev oscillators, experience a diminished nonlinear spectral broadening effect under RSA, which restricts spectral range and energy accumulation of pulses, adversely affecting pulse compression.

NPR/NPE, also known as polarization additive pulse mode-locked, have proven to be efficacious methodologies for engendering mode-locked pulse outputs. Simulation and experiment demonstrated that soliton pulse shapes remain invariant under intensity-dependent polarization rotation^113^. Subsequently, NPR-based Er-doped fiber laser (EDFL)^114^ was corroborated and an all-optical wavelength converter within a single semiconductor optical amplifier was introduced^115^. Masuda et al. improved the NPE technique using a ring cavity with a circulator, achieving ultrafast pulses of 39 fs^116^, successfully generating multi-wavelength comb-like waves in self-starting EDFL^117^. Simultaneously, the feasibility of dissipative soliton (DS) is indicated without the need for a band-pass filter and transient lasing mechanism interposed is delved into between stable single-pulse and NLP generation^118^ in all-normal dissipative (ANDi) Yb-doped fiber lasers (YDFL). Their investigation encompasses three distinct states of circulating single-pulse generation, analyzing spectral, temporal properties, and the compressibility of resultant pulses^119^. Concurrently, Peng et al. built an all-fiber NPR mode-locked laser with 693 fs and a bandwidth of 20 nm output of self-similar soliton pulses^120^. The tunable and switchable dual-wavelength ultrafast thulium-doped fiber laser (TDFL) harnessed the NPE effect to reduce wavelength-dependent losses, alleviating mode competition and enabling multi-wavelength mode-locked^121^, achieving the highest average power output of 174 mW for conventional solitons^122^. The nonlinear effect is the basis of pattern locking in the NPR structure. The presence of RSA leads to increased absorption of higher-intensity pulses, preventing the accumulation of sufficient nonlinear phase shifts, thereby diminishing the nonlinear modulation capability of the NPR setup and compromising the stability and maintenance of the mode-locked state.

#### Active mode-locking

Active mode-locking necessitates the incorporation of a modulation device within the laser resonator or the injection of an external light pulse to actively modulate the intracavity light waves, using radio frequency signals to obtain high-repetition frequency pulses and hold significant potential for communication applications. At present, modulator-based mode-locking and injection mode-locking technology are dominant in the field of active mode-locking^123^. Chiu et al. demonstrated mode-locking by exploiting cross-gain modulation effects in SMF through the backward injection of a dark-optical comb with a duty cycle of 94% and a pulse width of 25 ps at a high-repetition rate of 10 GHz^124^. This approach yielded the shortest pulses of 5.4 ps, ultimately generating modulated pulses with a pulse energy of 110 pJ and a compressed pulse width of 410 fs. Recently by injecting 1.5 μm master oscillator pulses into the laser cavity and inducing cross-phase modulation nonlinear effects, dispersion soliton resonance mode-locked (DSR-ML) lasers can be efficiently synchronized to a frequency standard, eliminating the necessity for intricate feedback loops or ps-scale pulse generators^125^. Exploiting the XPM nonlinearity, the DSR-ML laser generated 2 ns pulses with peak powers reaching 238 W, attaining stability that rivals or surpasses that of low-pulse-energy soliton ML fiber lasers, paving the way for utilization in applications necessitating precise timing. Modulator-based mode-locking relies on driving modulators with frequency signals to induce periodic amplitude or phase modulation within the cavity, including acousto-optic modulators, electro-optic modulators, and Mach-Zehnder interferometer^126^, as intracavity amplitude or phase modulators to enable the realization of active mode-locking. Mach-Zehnder interferometer is used in EDFL to generate femtosecond pulses with a pulse modulation width of ~500 fs^126^. A novel active mode-locking EDFL was validated using a LiNbO_3_ phase modulator, which generated 825 fs ultrafast pulses with a repetition rate of 10 GHz using a stretch time lens for spectrum broadening instead of fiber nonlinearity^127–129^, connecting external dispersion-shifted fiber can facilitate the compression of the pulse duration to 396 fs^128^. Despite the impressive gigahertz repetition rates of ultrafast pulses, and is suitable for applications that require high-repetition frequency pulses, such as high-speed communications, it still has great defects. The inclusion of a modulator complicates the cavity architecture and incurs higher costs. The pulse narrowing is limited by the response time of the modulator, which can impede the generation of stable femtosecond pulses, leading to more intricate structures. Typically, the pulse durations for MLFLs are on the picosecond scale, the existence of modulators diminishes the environmental stability of MLFLs.

#### Hybrid mode-locking

Hybrid mode-locking is a technique that amalgamates multiple mode-locked mechanisms within the cavity to enhance performance^130^. The strategy is to merge the ultrafast pulse generation ability of PML with the stability of active mode-locking, leveraging diverse mode-locking techniques to produce high repetition-rate and narrow-band pulses. Incorporating an active modulator within PMLFL facilitates the generation of regular pulse trains, producing high repetition-rate pulses at 9.2 GHz with an output power of 15 mW^131^ and pulse duration of 95 fs^132^. Employing hybrid mode-locking that ESA and ASA can further enhance pulse quality. For instance, combining NPE with single-wall carbon nanotube^133^, Sb_2_Te_3_^134^ has led to pulse durations of 90 fs^135^. NPE can produce short pulses with high peak power but is sensitive to environmental disturbances, whereas SESAM enables reliable self-starting and stable soliton pulse production, albeit with suboptimal pulse parameters. Combining both methods, hybrid mode-locking can achieve reliable ML with optimized pulse quality, high performance, and environmental stability. Based on NPE and SESAM^136^, pulse duration has been compressed to 41.9 fs^137^, while pulse energy has been increased to 163 nJ, comparable to solid-state lasers^138^. A 2 μm TDFL combining NALM and NPE^139,140^ is first proposed, achieving 49 fs pulses with an average power of 126 mW^141^, offering low noise and suitable for fiber comb-like construction. Wei et al. used MoS_2_-WS_2_ by magnetron sputtering, obtaining pulses of 152 fs^142^. Additionally, combining CNs with NPE in TDFL formed a saturable absorber with a large modulation depth, generating 560 fs pulses^142^. While this integrative approach offers considerable advantages for pushing the performance envelope, it increases the intricacy of the fiber cavity design, which is a deterrent for applications that require all-fiber systems.

### Challenges and solutions

In the realm of laser pulse research, the overarching objective for researchers is to obtain large pulse energy^143,144^, achieve high output power^143,145–147^, and narrow the pulse duration^148–152^. However, the existence of RSA increases the absorption of high-intensity pulses, preventing sufficient energy accumulation, which leads to inadequate pulse compression and increased pulse width, raising thresholds for mode-locking initiation, complicating the self-starting of mode-locking, and destabilizing ultrafast pulse generation. Additionally, the increased energy losses in high-intensity pulses result in reduced system output power and efficiency, particularly in applications requiring high-repetition rates^153–156^ or high-power output^157^. As can be seen in Table 1, beyond the exploration of FML dynamics in response to increased pump power, the laser resonator simultaneously sustains multiple solitons^158,159^ that coalesce into stable configurations via soliton interactions, yielding complex structures (HML^38,160^, soliton molecules^161–164^, soliton complexes^165^ and supramolecular structures^166^). By tuning elements such as gain^167–169^, dispersion^170–172^, polarization^173,174^, and other characteristics of the laser resonator^175,176^, the number of pulses, pulse spacing, relative phase, and other parameters of the multi-pulse can be controlled on demand. Nonetheless, excessive pump power can precipitate gain saturation in the gain fiber beyond critical thresholds, instigating severe pulse jitter during the mode-locked phase. The aforementioned techniques enable modulation of saturable absorbers modulation depth via waveplates or intracavity nonlinear effects to facilitate transitions between various pulsation states^177^, it is incontrovertible that such techniques are highly sensitive to thermal and mechanical stresses, presenting significant limitations^178,179^. In response to the instability of ML, two main solutions have emerged in recent years, one is the integration of all polarization-maintaining (all-PM) fiber into the laser architecture to obtain high-stability lasers, and the other is AML.

**Table 1:** Performance parameters of lasers doped with different rare earth elements

Method	Region	Gain medium	Repetition rate	Central wavelength	Laser output characteristics			Refs.	
					Pulse width	Output power	Pulse energy		
NPR	Solitons	Tm	11.6 MHz	1890 nm	350 fs	90 mW	7.8 nJ		^144^
NPE	Solitons	Tm	248 MHz	1950 nm	330 fs	-	74 pJ		^171^
NPE	Solitons	Tm	6.32 MHz	2003.2 nm	406 fs	78 mW	12.342 nJ		^172^
NPR	Solitons	Er	384 MHz	1561.8 nm	110 fs	207 mW	0.54 nJ		^155^
NPR	Solitons	Er	4.22 GHz	1603.31 nm	810 fs	8.95 mW	11.5 pJ		^44^
NPE	-	Er	100 MHz	1550 nm	200 fs	25 mW	70 pJ		^31^
NPE	-	Yb	54 MHz	-	110 fs	3 mW	60 pJ		^95^
NPR	Stretched Solitons	Er	37.8 MHz	1550 nm	55 fs	56.4 mW	1.5 nJ		^148^
NPR	Stretched Solitons	Er	37.9 MHz	1551 nm	80 fs	14.6 mW	0.47 nJ		^158^
NPR	DSs	Er	28.6 MHz	1550 nm	78.9 fs	188 mW	4.6 nJ		^159^
NPR	DSs	Er	36 MHz	1600 nm	57.6 fs	47 mW	1.3 nJ		^175^
NPR	DSs	Er	11.6 MHz	1599.6 nm	156 fs	68.6 mW	6.15 nJ		^147^
NPR	Self-similar Solitons	Er	37 MHz	1550 nm	37 fs	-	1.3 nJ		^149^
NPE	Stretched Solitons	Er	205 MHz	1530 nm	93 fs	70 mW	-		^41^
NPE	Stretched Solitons	Er	90.5 MHz	1550 nm	90 fs	180 mW	77 pJ		^153^
NPE	Stretched Solitons	Er	301.3 MHz	1550 nm	90 fs	125 mW (CW) 69 mW (ML)	-		^177^
NPE	Stretched Solitons	Er	11.3 MHz	1560 nm	180 fs	30 mW	2.6 nJ		^157^
NPE	Stretched Solitons	Er	12 MHz	1560.25 nm	84 fs	30 mW	2.5 nJ		^97^
NPR	Self-similar Solitons	Yb	616 MHz	1060 nm	98 fs	450 mW	0.73 nJ		^151^
NPE	Stretched Solitons	Yb	66.1 MHz	1060 nm	65 fs	28 mW	0.4 nJ		^152^
NPE	Stretched Solitons	Yb	18 MHz	1050 nm	90 fs	85 mW	4.5 nJ		^94^
NPE	Stretched Solitons	Yb	58 MHz	1030 nm	~25 fs	130 mW	2.3 nJ		^150^
NPE	DSs	Yb	71.3 MHz	1032 nm	76 fs	259 mW	3.6 nJ		^176^
NPE	DSs	Yb	20.54 MHz	1030 nm	150 fs	17.5 mW	0.85 nJ		^180^
NPE	DSs	Yb	312 MHz	1060 nm	118 fs	205 mW(max)	0.56 nJ		^154^
NPE	DSs	Yb	70 MHz	1050 nm	80 fs	2.2 W	31 nJ		^145^
NPE	DSs	Yb	73.46 MHz	1041 nm	102 fs	9 W	122 nJ		^143^
NPE	DSs	Yb	58 MHz	1033 nm	182 fs	16 W	0.28 μJ		^146^
NPE	Solitons	Yb	162 MHz	1030 nm	~100 fs	29 mW	145 pJ		^156^

*CW* continuous wave

**Fig. 4:**
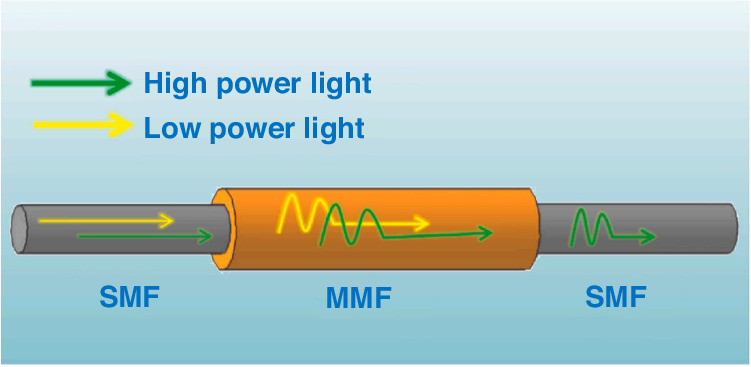
**The schematic of SMF-GIMF-SMF**. Adapted with permission from ref. ^185^ ©Elsevier

The fundamental principle of all-PM mode-locking is to replace conventional SMF with PM fibers, countervailing the birefringence effects inherent in PM fibers using splice-crossing methodologies, which diminishes self-focusing positions, mode dispersion, and moduli of guided waves. Advancements in 2017 by Szczepanek et al. involved precision splicing of PM fibers at varying angles, enabling the laser to operate with pulse durations of 150 fs, repetition rate of 20.54 MHz, and maximum average output power of 17.5 mW, yielding pulse energies of ~0.85 nJ^180,181^. In 2018, Li et al. achieved laser output with a repetition rate of 111 MHz and pulse energy of 0.47 nJ by meticulously setting the welding angle, with timing jitter and relative intensity noise measured to be 6.41 fs and 0.0052%, respectively^182^. Xu et al. utilized graded-index multimode fibers (GIMF) to produce pulses of 980 fs^183^, while Wang et al. used GIMF as a compressor to generate pulses of 416 fs, the structural diagram is shown in Fig. 4^184,185^. A pioneering SMF-GIMF-SMF structure was introduced in 2018, splicing GIMF between SMFs to achieve 387 fs stretched soliton pulses in the ring cavity^59,186,187^. Furthermore, iii-nitride semiconductors InN and chalcogenide glass As_40_Se_60_ have been explored, achieving pulses of 55 fs in EDFL^188,189^. Although experiments have demonstrated that cross-splicing can effectively achieve ML, the presence of solid-state devices within the cavity presents challenges in generating shorter pulse durations.

In response to these limitations, the concept of automatic control for AML has garnered significant interest within the photonics community. The basic idea of AML is to adjust the external voltage to change the control on the Upper computer with algorithms. By monitoring the changes in the intracavity polarization state, the information is instantaneously fed back to the computer for automatic adjustment, achieving stable pulse output^190^. The core of AML is a closed-loop feedback system, which is composed of an acquisition, calculation, and control module. The acquisition module is responsible for recording the output data of the laser and usually requires the use of modules or instruments that can obtain the time-frequency information, such as analog-to-digital conversion chips (ADC), oscilloscopes, spectrometers, spectrometers, etc., which can be used separately or simultaneously according to the needs of data analysis. In recent years, researchers have used Time-Stretched Dispersive Fourier Transform (TS-DFT) technology^191^ combined with a high-speed oscilloscope as a sampling module to observe the spectral width and shape of the ultrafine laser. The computing module is responsible for processing and analyzing the data, which usually requires the use of a device with strong computing power and fast computing speed^56,173,192^. The control module is responsible for the accurate regulation of the laser state and for formulating the next adjustment of the control modules. It is worth noting that the response bandwidth of the control module is determined by the selected device bandwidth.

## Automatic mode-locking technology

Integrating hardware to detect and extract the relevant information of the parameters in the cavity (such as polarization states^193^, energy jitter^194^, pump power, EPC, and pulse duration parameters, etc.), combining with the control algorithm, the pulse state of the laser is automatically identified and then fed back to the electronic devices to design a feedback circuit enables the system to swiftly transition into ML^195^. The polarization control algorithm employed must offer a high degree of speed and precision, robust search capabilities, and rapid convergence to ascertain optimal solutions. Currently, the polarization control algorithms include traversal algorithm^196^, evolutionary algorithm(including Genetics algorithm)^50,57,58,197^, HLA^192^, deep learning^52,54,198–202^, gradient algorithm^59^ simulated annealing algorithm^60–62^, etc. The algorithms are capable of enhancing the speed, output pulse characteristic, and accuracy of polarization control to varying extents.

### Traversal algorithm

**Fig. 5:**
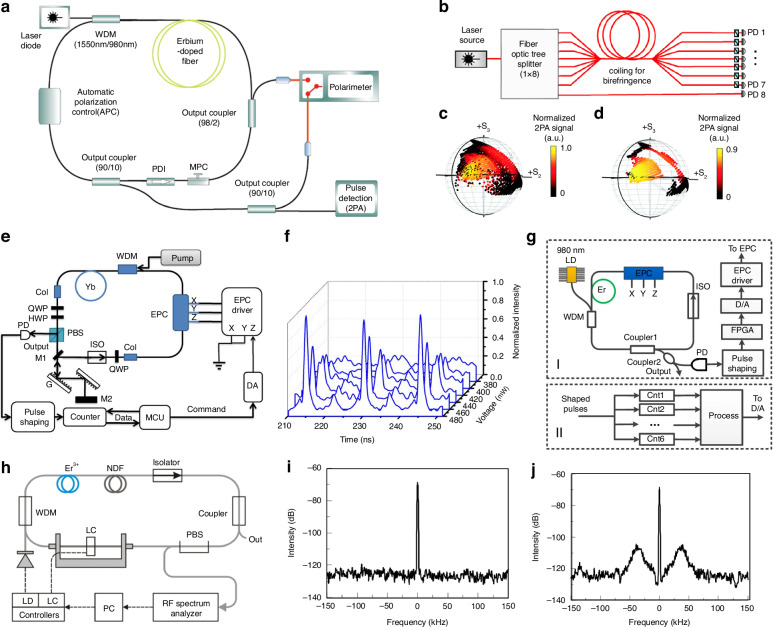
**Implementation overview based on traversal algorithm with diverse devices**. **a** Experimental device. **b** Schematic diagram of an all-fiber amplitude division polarimeter (PD: photodiode for PD1 to PD7 with front polarizer). **c** When the TPA signal exceeds the mode-locked threshold, color coding is displayed on the Poincaré sphere. **d** after the irregular pulse numerical filtering. Adapted with permission from ref. ^196^ ©Springer Nature. **e** Experimental setup. **f** Evolution of the pulse profile with variation of the input voltage at the EPC. **g** The optimized experimental setup. Adapted with permission from ref. ^195^ ©Elsevier. **h** Experimental device. **i** RF inter-mode beat spectrum of the most stable and (**j**) less stable FML regime at around 8 MHz. Adapted with permission from ref. ^193^ ©Springer Nature

Utilized computer-controlled PCs and high-speed all-fiber division-of-amplitude polarimeter as shown in Fig. 5b, Hellwig et al. achieved, for the first time in 2010, successful characterization of MLFL based on NPR as illustrated in Fig. 5a^196^. The characterization of the laser involves establishing a mapping relationship between polarization states and system parameters, including pulse duration, central wavelength, and average output power. The initial polarization state is controlled using conventional manual polarization controllers (MPCs) and the automated polarization scanning results on the Poincaré sphere are depicted in Fig. 5c, where pulsating regions correspond to polarization areas with strong TPA signal values. Utilizing a rapid-measurement device predicated on TPA to enable swift scanning across a multitude of data points, however, pulsating states also encompass other states. Numerical filtering of the scanning results yields the polarization region corresponding to the mode-locked zone, as shown in Fig. 5d. While complete control over the four-dimensional parameter space allowed for better optimization of system parameters (pulse duration, central wavelength), long-term stable ML remains challenging.

Pulse counting methods and feedback mechanisms were employed for the first time, as shown in Fig. 5e, tuning the input voltage of the EPC for precise control^56,193^. They employed EPC for fine-tuning polarization states, using optical nonlinearities to control laser polarization, as shown in Fig. 5f enabling the laser to traverse different polarization states with varying input voltage. Voltage adjustments led to observable spectral broadening, pulse width modulation, and carrier-envelope-phase (CEP) frequency shifts, enabling sensitive control over the EPC. The determination of mode-locked states relied on the pulse counting with MCU continuously controlling the EPC to adjust the intracavity polarization states, the EPC stops adjusting upon MCU recognition of the current state as ML. Figure 5f illustrates the variation of pulse characteristics in response to changes in the scanning voltage, capturing the self-starting process from model-unlocking to model-locking. The output voltage of the data processing module fluctuates between 390 and 440 mV, the change in the EPC driving voltage of 1 mV resulting in the linear frequency shift of CEP by 2.52 MHz, corresponding to π/16 rotation of the intracavity polarization, showcasing the substantial potential for temporal and spectral control. However, manual polarization control using intracavity waveplates remained necessary if ML was not realized within the EPC voltage range.

In 2015, stable repetition-rate control of the EDFL mode-locked oscillator was achieved utilizing the aforementioned apparatus^203^. Under the influence of low feedback voltage applied to EPC, the standard deviation and linewidth of the repetition rate are 1.4 MHz and 1.7 MHz, respectively, validating the capability to realize highly integrated, low-noise optical frequency combs. Further advancements in 2017 involved the precise intracavity polarization state adjustment using EPC, with corresponding driving voltage recordings during ML, as shown in Fig. 5g^193,204^. This database aids in reducing setup time and temperature dependence of mode-locked, demonstrating repeatability and stability over a broad temperature range.

On the same algorithmic basis, Radnatarov et al. analyzed the intermodal beat spectrum of output radiation using liquid crystal (LC) to determine the optimal wave delay, controlled the single-chip LC within range of 0–0.6λ under driving voltage of less than 4 V to optimize the recovery of the failed state according to the mode-locked conditions of the emission spectrum, as shown in Fig. 5h^98,193^. The algorithm could discern the mode-locked state based on the emitted spectrum, effectuating initiation and optimization. As Fig. 5i illustrates, the absence of sidebands in the RF spectrum correlates with the highest amplitude of RF peaks, denoting the most stable FML state. However, the FML stability regime declines due to increase in pump power and the amplitude reduction caused by LC birefringent displacement, resulting in additional satellite peaks adjacent to the main peak in the RF spectrum as shown in Fig. 5j and multi-pulse ML, respectively.

**Fig. 6:**
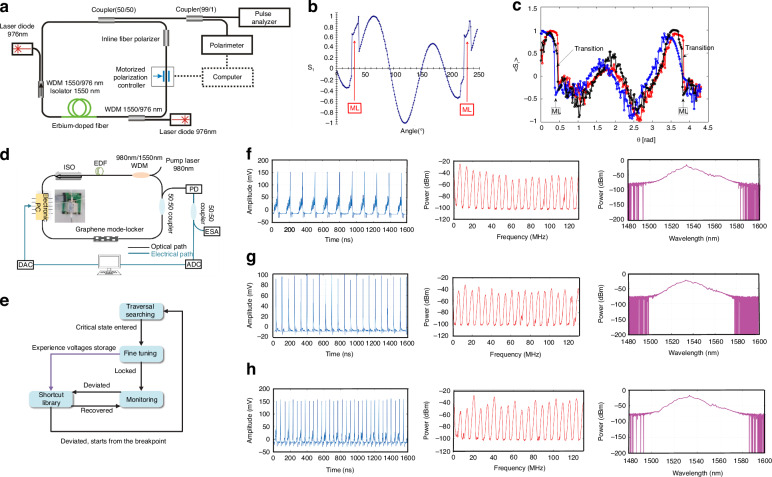
**Description of experimental result based on traversal algorithms with different structures**. **a** Experimental device. Adapted with permission from ref. ^193^ ©Springer Nature. **b** Simulation results. Adapted with permission from ref. ^206^ ©Journal of Visualized Experiments. **c** Experimental results for the averaged first Stokes parameter obtained once a steady state is reached: unperturbed case (red), perturbation before EDFL (blue), and perturbation after EDFL (black). “ML” indicates the attainment of stable mode-locked operation. Adapted with permission from ref. ^193^ ©Springer Nature. **d** Experimental device. **e** Simplified traversal algorithm. **f** Time-domain/Frequency-domain /optical spectrum of FML state. **g** Time-domain/Frequency-domain/optical spectrum of second-order HML state. **h** Time-domain/Frequency-domain /optical spectrum of third-order HML state. Adapted with permission from ref. ^207^ ©Optical Society of America

Similar to ref. ^56^, MCU ran the traversal algorithm centered on pulse counting to search for the mode-locking region, Li et al. used two EPCs to achieve AML with repetition rate of 6.238 MHz^40^. The laser automatically realizes ML within 90 s and continuously monitors output pulses for loss of lock recovery and parameters saved in electrically erasable programmable read-only memory for rapid realize ML next time. However, the current system is limited to identifying FML and potentially be adapted to search for HML after modifying the pulse counting rules, while searching for QS and QML states is beyond its capability. To distinguish the mode-locking mechanism, by considering the interaction of polarizers, waveplates, and intracavity NPR, Oliver et al. adopted EPC and feedback loops as shown in Fig. 6a, achieving AML by detecting discontinuous jumps of polarization states^193,205,206^. The experimental and theoretical conclusions are consistent as shown in Fig. 6b, c, that is, when the laser state changes, a discontinuous jump will be detected, which proves the feasibility of single-waveplate mode-locking. However, the study does not address the potential impact of changes in the polarization alterations of output laser when transitioning into other pulsing regions (the HML, the QS) on the accurate identification of the FML region.

**Fig. 7:**
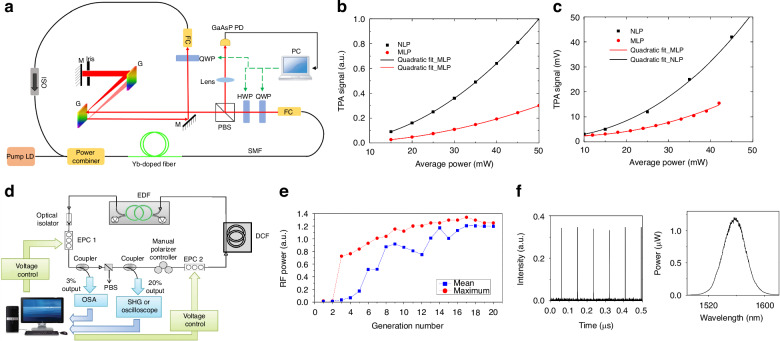
**Description of experimental result based on traversal algorithms and GA**. **a** Experimental setup. Quadratic relationship between theoretical (**b**) and experimental (**c**) TPA signal strength and average power for NLP and FML modes. Adapted with permission from ref. ^193^ ©Springer Nature. **d** Experimental setup. **e** Convergence of the best (red dot) and average (blue square dot) fitness EA defined by average power of SHG value. **f** The temporal trace of the output intensity and optical spectrum of the fittest candidate solution corresponds to the optimized pulsed laser regime. Adapted with permission from ref. ^193^ ©Springer Nature

Different from ref. ^98,205^, Pu et al. leveraged graphene as both a polarizer and saturable absorber to achieve AML (FML, second/third-order HML) in Fig. 6d^207^. Utilized traversal algorithm (the algorithm’s structure is shown in Fig. 6e), the database is established by linking voltage levels to corresponding pulse states with ADCs utilized for data collection and fast Fourier transform (FFT) techniques employed to analyze higher-order harmonics. For analysis of higher-order harmonics is shown in Fig. 6f–h, it is noted that the *n*th harmonic of HML displays the greatest amplitude at its *n*th spectral line, but the spectrogram cannot be used as a basis to distinguish the state of the output pulse. Simultaneously, Wu et al. used EPC in dispersion-mapped and ANDi fiber laser to realize AML (NLPs, FML), further reducing the tracking and formation time of target pulses^193,208^. The experimental settings are shown in Fig. 7a. The distinction between NLP and FML states was made, with theoretical simulations (Fig. 7b) and experimental measurements (Fig. 7c) conducted to compare the TPA signal intensity induced by NLP and FML, respectively, which indicated that the TPA signal elicited by NLP is significantly more intense than that by FML. The study also proposed a feedback mechanism by GaAsP photodiode to effectively auto-tune between different mode-locked states. However, other pulsation mechanisms inducing TPA signals are not discussed.

The traversal algorithm is relatively straightforward and is directly applied to various conditions, ensuring global optimal solutions through exhaustive enumeration of all possible parameter combinations. However, as parameters increase, computation time and resource requirements grow exponentially, resulting in inefficiency and unsuitable for real-time applications requiring rapid adjustments. Consequently, the traversal algorithm has gradually been supplanted by other algorithms with superior performance.

### Evolutionary algorithm (EA)

As a robust optimization strategy with broad system applicability, EA comprises different heuristics for optimization, not limited to a single algorithmic structure, surpassing traditional traversal algorithms and exhaustive search methods due to intrinsic capacities of self-organization, adaptation, and learning in handling complex problems. Genetic algorithm (GA), a fundamental variant of EA, was first introduced in 1975^209,210^, utilizing fitness functions and system parameters to optimize. Local optima are avoided, endowing GA with inherent advantage and making it widely utilized in AML techniques within the domain of ultrafast photonics.

To effectively enhance single-pulse energy, mitigate and suppress multi-pulse instability, and increase system stability and accuracy, GA is employed to optimize multiple NPR filters^211^. For the first time, two EPCs and one MPC were used to adjust the polarization state within optical cavity as shown in Fig. 7d, and used the fitness value to optimize the six voltages on the EPC^193,212^, improving the pulse energy and realizing AML. They propose a fitness function based on the second harmonic generation (SHG) signal intensity generated within nonlinear crystal BaB_2_O_4_ at the laser output. In the control process, the strong SHG pulse signal can be produced by FML and is amplified by optimizing the phase adjustment of the drive voltage opening to control EPC. However, QML will occur during the optimization process, which could not be differentiated from FML and HML using this function, complicating the assurance of successful optimization in every instance and consistent avoidance of QML, as shown in Fig. 7e. To solve this problem, they proposed a new fitness function to obtain the spectral component intensity of the free spectral range by combining FFT of the time-domain signal for searching the *n*th HML pulse state^213^. The convergence process of the fitness function is shown in (2)^213^, which ensures that mode-locked states each time as presented in Fig. 7f.

2 $$M=\frac{A({nFSR})}{\frac{{\sum }_{i=1}^{n-1}A\left({iFSR}\right)+{\sum }_{i=1+1}^{2n-1}A\left({iFSR}\right)}{2n-3}+C}$$

where *A* is the amplitude of the repetition frequency of order *n*, and *C* is the constant of floor noise. The research is a huge six-dimensional parameter space and complex algorithm structure, which takes about 30 min to achieve ML^213^. The primary stems from the slow data acquisition of experimental setup and computational intricacy inherent in the search processes. Swiftly achieving mode-locked poses a significant challenge that early GAs must confront.

**Fig. 8:**
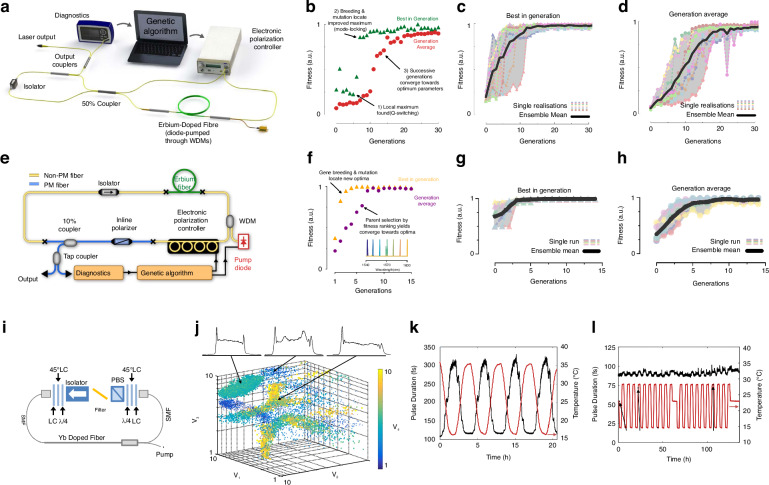
**Description of experimental for different fitness functions based on GA**. **a** Experimental setup. **b** convergence of fitness for a single realization, **c** the convergence of maximal, and **d** average fitness values over ten realizations. Adapted with permission from ref. ^193^ ©Springer Nature. **e** Experimental equipment. **f** fitness evolution (inset: self-tuned spectra showing tuning range). **g** Self-tuning characteristics with targets parameter: evolution of **g** “best” fitness and **h** “average” fitness. Adapted with permission from ref. ^209^ ©Optical Society of America. **i** The experimental setup with liquid crystals. **j** States are found by scanning over all available voltages. Pulse duration as a function of environmental temperature in **k** standard oscillator and **l** LC stabilized oscillator (minimizing spectral error and power error). Adapted with permission from ref. ^215^ ©Optical Society of America

Considering the complexity of multi-parameter optimization for stable mode-locked, the operating states of the laser are modulated by fine-tuning the nonlinear transfer function of the absorber through EPC and pump power, Woodward and Kelleher employed GAs for self-optimization of ultrafast pulses, achieving stable single-pulse mode-locked with NALM architecture as shown in Fig. 8a^193,214^, probing 2D section of four-dimensional polarization parameter space. With the pump power maintained at a constant level, the fitness function quantifies laser performance by assessing output attributes such as a single realization of the evolutionary process and 10 results of this repeated experiment as illustrated in Fig. 8b–d, offering a quantitative measure of performances. The average optimization process takes about 20 generations (Fig. 8c) and is usually completed within 30 min. The convergence time mainly depends on the EPC, the pump diode controller, etc. In the subsequent phase, combined with automatic pump power control to optimize self-tuning birefringent filter control for auto-pulsing, they utilized EPCs and inline fiber polarizers as shown in Fig. 8e to adjust the phase bias of the birefringent filters, achieving stable and tunable QS state^209^. The system allows for higher transmission of high-intensity laser, producing QS pulses over 55 nm with a variable repetition rate of 25 kHz and identification of the globally optimal setting. They achieved a stable single-pulse ML and observed that the light traversing the PM fiber post-polarization retained linear polarization along the slow axis due to the selective power transmission properties of the polarizer. Moreover, the comparisons between Fig. 8b–d and Fig. 8f–h reveal that the new fitness function exhibits enhanced convergence properties.

Winters et al. extended the operational range of phase retarders to a full 2π, thereby enabling coverage across the entire four-dimensional parameter space^215^. The setup is depicted in Fig. 8i. Figure 8j illustrates the search of FML states while scanning four LCs. Global optimization excels when the laser’s state significantly diverges from the target or requires an exhaustive search, while the local search algorithm rapidly converges to the solution when the laser operates near the desired state. Employing a hill-climbing algorithm to deal with the separation of the desired state within 30 s, substantially enhancing the stability of the output pulse duration, as illustrated in Fig. 8k, l and the degree of similarity between the theoretical and experimental spectra is quantified using the coefficient of determination. Although the method facilitates mode- locking within 90 s, drastically reducing the initial mode-locked time and recovery time, the operational duration is constrained by the response rate of LC (~2 s). It is important to note that the selection of fast-response components such as electro-optic modulators is crucial to diminishing the response time.

**Fig. 9:**
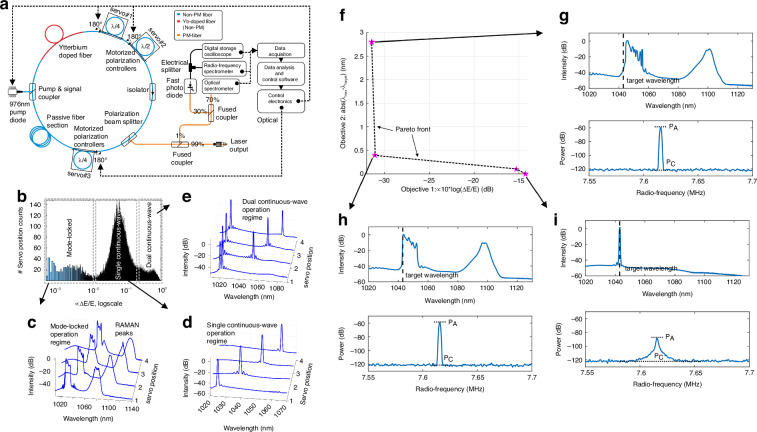
**Description of experimental results by using pulse energy jitter and wavelength as optimization objectives**. **a** Experimental setup. **b** The amplitude jitter of the full polarization scanning pulse is represented by distribution histogram. **c** The laser works at FML range below the jitter value. **d** The laser works in a single CW state at the maximum peak of the histogram, and the two-objective optimization is achieved. **e** Laser lines with multiple CW states appear in the spectrum at large jitter values. **f** The distribution of mechanisms on the objective value map. **g** The system of operations is consistent with objective 1 but not with objective 2. **h** The operational system is in line with objectives 1 and 2. **i** The operational regime is consistent with objective 2, but not with objective 1. Adapted with permission from ref. ^193^ ©Springer Nature

Different from the studies mentioned above, Ryser et al. combined EPC with GA for the regulation of the system as shown in Fig. 9a^193,194^. The laser system is augmented with three motorized PCs and an inline monitoring system, which employs pulse energy jitter and wavelength as optimization targets. Analyzing the three-dimensional radio frequency spectrum graph at a pump power of 350 mW reveals a histogram depicting the distribution of pulse amplitude jitter as shown in Fig. 9b. As Fig. 9e displays, the laser, in a dual CW state, is tunable between 1.4 THz and 13.5 THz. Figure 9c shows the laser operating at a jitter value of ~0.01 in the FML state. At the jitter value proximate to 0.1, the laser works in a single CW state as delineated in Fig. 9d. The study achieves multi-objective optimization, the compound fitness function is formulated from pulse amplitude jitter (target 1) and the deviation of the measured wavelength from the target wavelength at maximum intensity (target 2). Figure 9f maps the distribution of various states in the multi-objective optimization space. Figure 9g presents a state with low jitter values satisfying target 1 but not target 2, while Fig. 9h delineates the ideal operational regime for meeting both targets effectively. Finally, Fig. 9i depicts a state with high jitter, which meets target 2 as the obtained wavelength closely matches the target wavelength, but fails to fulfill the requirements of target 1.

**Fig. 10:**
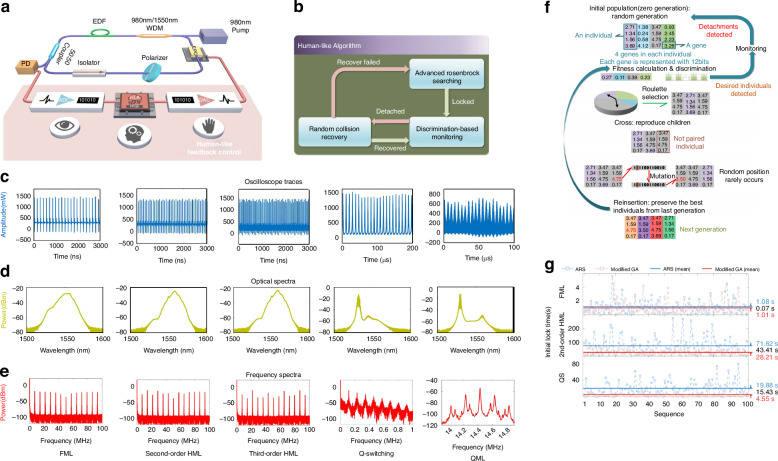
**Description of experimental results of different algorithms for similar devices**. **a** Experimental setup. **b** Structure diagram of HLA. **c**–**e** The operation regimes, from left to right, comprise FML, second-order HML, third-order HML, QS, and QML, with each column depicting oscilloscope traces (top row), optical spectra (middle row), and frequency spectra (bottom row). Adapted with permission from ref. ^192^ ©Optical Society of America. **f** Schematic of the proposed modified GA. **g** The modified GA and the ARS are compared in terms of their time-consumption performance, with the squares on the dashed lines representing the measured initial lock times and the solid lines denoting the average time consumption. Adapted with permission from ref. ^193^ ©Springer Nature

Diverging from the aforementioned studies, Pu achieved a groundbreaking advancement by introducing the HLA, which enabled the rapid realization of FML, HML, QS, and QML without necessitating experimental apparatus reconfiguration as illustrated in Fig. 10a^192^. The HLA integrates an advanced Rosenbrock search algorithm with a stochastic collision recovery process and region-specific identification mechanism to expedite response times in Fig. 10b, enabling microsecond-scale transitions between operational states. Under pump power of 600 mW, the measured oscilloscope traces and optical/frequency spectra for FML, second and third-order HML, QS, and QML are presented in Fig. 10c–e. Close examination of the spectra from the second and third-order HML regimes reveals that the second-order and third-order fundamental spectral lines exhibit significantly higher intensity compared to the other spectral lines, confirming the efficacy of the proposed HML objective function. The spectrum of the QS regime reveals a repetition frequency of ~115 kHz for the QS pulses, which is considerably lower than that of the mode-locking mechanism, with a spectral width much narrower than that of the mode-locking mechanism. The QML envelope frequency measures 200 kHz, while the carrier fundamental corresponds to the second-order of the fundamental repetition frequency. HLA amalgamates heuristic approaches with computational swiftness and precision, significantly refining the initial mode-locked duration and achieving a recovery time of 14.8 ms.

In subsequent enhancement, they improved GA, terminating the algorithm upon reaching the target state, and reducing the time consumption of the system as depicted in Fig. 10f^193,210^. Compared to the Rosenbrock search technique modified GA accelerates the search for second-order HML and QS states by ~2.5 times and 4.5 times, respectively. The laser averagely locks into the FML in a mere 1.01 s, while the average search times of the second-order harmonic modulation-locked state and the QS state is 28.21 s and 4.45 s, respectively. These results showcase the formidable potential of the modified GA in the realm of AML. Further advancing the field in 2020, Pu et al. employed a time-stretch real-time pulse controller to achieve AML with an enhanced compression ratio, pulse repetition rates, and signal-to-noise ratio (SNR) over conventional technologies^173,210^. The system also reveals complex and repeatable transition dynamics between different pulse states, is able to program the spectral shape of femtosecond pulses to be hyperbolic secants or triangles, with spectral widths that can be tuned between 10 and 40 nm and resolution of about 1.47 nm. Enabling TS-DFT, complex and reproducible dynamics of pulse state transitions are observed. These dynamics are unattainable with conventional mode-locked lasers.

Girardot et al. implemented GA to realize mode-locking in fiber laser featuring four-parameter NPE and normal dispersion^191^. By fine-tuning intracavity PC parameters and employing the TS-DFT, they efficiently optimized the performance of MLFL, enabling precise spectral width adjustments. The system swiftly identifies a predefined optimal laser state and the average high-resolution spectrum is recorded. Manipulating the intracavity polarization states can completely control the nonlinear transfer function, which produces more robust DSs in a normally dispersive cavity compared to those in anomalous dispersion.

In the pursuit of algorithmic enhancement, Zhu et al. introduced a hybrid algorithm that combines GAs with particle swarm optimization^216^, enabling the precise manipulation of temporal intervals between multiple solitons, providing a viable approach for probing the nonlinear soliton dynamics within optical systems and for the optimization of ultrafast laser performance by adjusting EPC. Moreover, the combination of GA and machine learning strategy ensures smooth navigation towards the global maximum, enabling the maintenance of high-energy pulse states and facilitating rapid state recognition and optimization, circumventing certain limitations of traditional feedback controls.

EA/GA possesses global search capabilities, effectively avoiding local optima through the simulation of natural selection and genetic variation, demonstrating superior global search abilities that can adapt to different mode-locking mechanisms and parameter variations and handling complex nonlinear problems. Although their global search capabilities are robust, the convergence speed to the optimal solution is relatively slow, potentially requiring extended time. The performance of the algorithm is sensitive to the settings of parameters such as selection, crossover, and mutation, necessitating repeated experimentation to optimize parameters, exhibiting high complexity, making implementation and debugging particularly intricate, especially in multi-dimensional parameter spaces requiring substantial computational resources.

### Machine learning

Machine learning has been revolutionizing the fields of science and technology in recent years^217^, with machine learning providing the most captivating and successful mathematical framework for model inference to date. Intelligent algorithms applied to intelligent mode-locked lasers have evolved from dependent exhaustive search and optimization techniques to machine learning algorithms^52,201,217,218^ driven by machine learning algorithms (the ring search algorithm^219^, recurrent neural network (RNN) algorithm^199^, Deep Q-Network algorithm^220,221^).

**Fig. 11:**
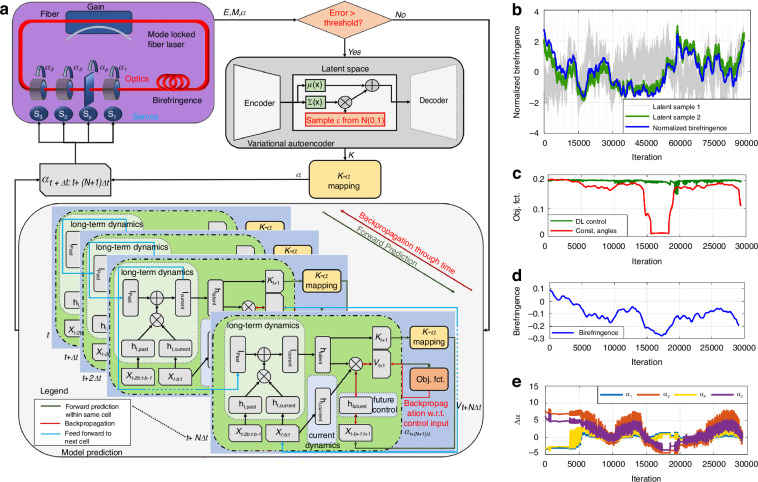
**Description of integrating machine learning into mode-locked control**. **a** machine learning control diagram. **b**–**e** When the birefringence changes randomly, DL-MPC again makes the objective function of the system stable at a high level. Adapted with permission from ref. ^193^ ©Springer Nature

Fu et al. first characterized the birefringent properties of MLFLs using machine learning and sparsity in numerical simulations^219^. The time series of the objective function is generated through the alteration of waveplate and polarizer angles, subsequently transformed into a spectrogram and subjected to dimensionality reduction via singular value decomposition, ultimately labeled with the corresponding effective birefringence, classification of the test measurement parameters is executed utilizing a sparse search algorithm (*L1*-norm optimization) to accurately identify the birefringence characteristics present in fiber lasers. The well-aligned case yields a birefringence recognition (classification) rate of 98%, whereas the misaligned case results in an 88% recognition rate^219^. Simultaneously, the theory developed is demonstrated on an NPR-based laser using waveplate and polarizer angles to achieve AML^201^. This is the first demonstration in the optical laser context of the integration of an adaptive extremum-seeking controller with a machine learning-based birefringence classifier has enabled researchers to formulate a self-adjusting design strategy for MLFLs based on numerical simulations. In 2018, Brunton et al. advanced their design by developing a mode-locked laser system underpinned with deep learning and model predictive control (DL-MPC) algorithms as shown in Fig. 11a^193,199^. As shown in Fig. 11b, although birefringent varies randomly with the number of cavity round-trips or iterations, it can also be correctly tracked or inferred, which proves the efficiency in recognizing laser birefringent. Through the comparative analysis of the system performance of MLFL with and without DL-MPC, as shown in Fig. 11c, it is demonstrated that DL-MPC enables MLFL to operate at peak performance without exiting mode-locking due to variations in birefringence. Figure 11d, e illustrate the specific changes in birefringence and the evolution of the polarizer and waveplate induced by DL-MPC. At the cornerstone of the algorithmic framework lies the predictive modeling module, when the discrepancy between the predicted laser state and the actual laser state exceeds a certain threshold, the neural network infers the birefringence of the system and maps it to control input parameters to maintain stable pulse output.

**Fig. 12:**
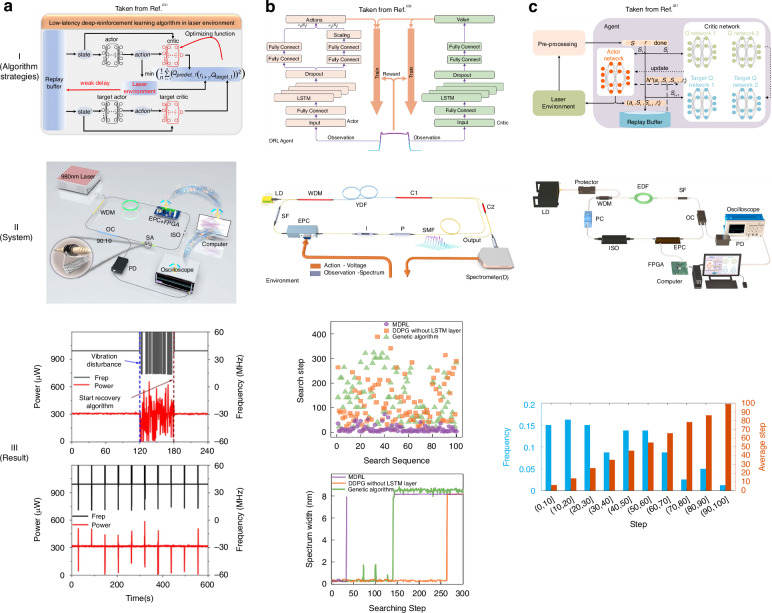
**Description of integrating machine learning and different strategies into mode-locked control**. **a**: (**I**) Algorithm structure based on DDPG strategy. (**II**) Experimental equipment. (**III**) Top: Variations in the output frequency of the laser and power during the application vibration to the laser and the activation of the recovery algorithm. Low: The output speed and power changes of the system within 10 min when the vibration is 1.5 s per min and the mode-locked recovery algorithm is running all the time. Adapted with permission from ref. ^231^ ©Chinese Laser Press. **b**: (**I**) Algorithm structure based on MDRL strategy. (**II**) Experimental equipment. (**III**) Total search step from 100 random initial states to the ML using MDRL (purple solid circle), DDPG (orange solid square), GA (green solid triangle), and search stability test with MDRL (purple), DDPG (orange), GA (green). Adapted with permission from ref. ^233^ ©Chinese Laser Press. **c**: (**I**) feedback control model. (**II**) Experimental equipment. (**III**) the results of 80 mode-locked tests. Adapted with permission from ref. ^221^ ©Elsevier

For control applications, various machine strategies have been developed to stabilize optical systems^199,217,219,222–224^. Deep reinforcement learning (DRL)^225–227^, one of the most successful machine learning architectures, has gradually evolved in recent years. As a rapidly growing branch of machine learning, DRL is a goal-oriented algorithm, learning from interactions with the environment and guided by objectives^228,229^. Through trial-and-error search, DRL models perceive the state of the environment and take corresponding actions to obtain the best immediate or delayed rewards. This sets DRL apart from the other two mainstream machine learning paradigms, namely supervised and unsupervised learning^230^. Yan et al. created the deep-reinforcement learning algorithm with a low latency (DELAY) algorithm (DRL approach rooted in a Deep Deterministic Policy Gradient (DDPG) strategy) and successfully applied it to the ultrafast fiber laser (UFL) system shown in Fig. 12a(II)^231^. The architecture of the DELAY algorithm, as depicted in Fig. 12a(I), mainly includes dual actor neural networks that devise strategies for adjusting the intracavity lasing networks that assess the effect of the actor networks. The experimental results in Fig. 12a(III) show that the DELAY algorithm achieves an average recovery time of 1.948 s, with the quickest recorded time being 0.472 s in the vibration test. This algorithm maintains consistent recovery times, approximately within 2 s, across different temperature conditions, illustrating its robustness. In addition, Fig. 12a(III) also shows the changes in system speed and output power within 10 min during the experimental test. Moreover, the system communicates with the MCU/oscilloscope for remote control and monitoring capabilities, ensuring prompt AML state recovery and robustness in remote operations and diverse environments. This capability also allows a single computer to remotely AML control across multiple laser systems, significantly enhancing the management of cascaded systems. It is noteworthy that, with the acceleration provided by GPUs, the entirely data-driven approach can significantly reduce computational complexity, enabling the rapid execution of automated inverse design processes in less than 1.3 h^232^.

DRL can enhance the performance of AML techniques by combining them with other multiple models. Li et al. have integrated DRL with long short-term memory (LSTM) networks to propose mode-locked operating mode search (MDRL) and the mode-locked state prediction (MSP) based on spectrum series learning^233^. The feedback control system is illustrated in Fig. 12b(II). MDRL network is utilized to learn and quantify the differences between various mode-locked states, as depicted in Fig. 12b(I). Through the extraction of correlated features from the time series and the incorporation of spectral information, a physical mapping is established between the input spectrum and the EPC state of the output, significantly enhancing the search efficiency. Experimental results show that the MDRL algorithm can achieve a stable mode-locked state within 690 ms, which is an order of magnitude faster than conventional GA methods. What is more, the pump power prediction error is less than 2 mW, ensuring precise locking across multiple operating states. To observe the spectrum evolution during the process of mode-locked search, we can observe the spectrum distribution of each search step in the initial state. The spectral width variation depicted in the lower part of Fig. 12b(III) demonstrates that MDRL can rapidly reach a stable mode-locked state (purple line), while GA cannot ensure the stability of the searched mode-locked state (green line). Although strict termination conditions enable GA searches to attain a stable mode-locked state, this approach necessitates additional search steps.

The average search time is one of the comprehensive metrics for evaluating the efficiency in achieving mode-locked, considering the inherent variability in the laser environment and the potential bias introduced by comparing the duration of the single search iteration from the random initial state to the FML condition. Li et al. have enhanced the capabilities of DRL by incorporating the Soft Actor-Critic (SAC) strategy, as depicted in Fig. 12c(I) and the system like Fig. 12c(II)^221^. The fundamental framework of the SAC comprises a policy network, two Q networks, and two target Q networks. the Q networks provide assessments of the soft state values, while the target Q networks evaluate the soft state-action values to facilitate the updating of the policy network. The double Q network mitigates the risk of overestimation caused by maximization via selecting the minimum Q value. The policy network generates an action distribution and adjusts the laser based on probabilistic sampling. Following each exploration episode, the actions, states, adjusted states, and rewards are meticulously recorded and transferred to an experienced pool. Upon accumulating sufficient experience, a random batch of state transitions is extracted from the experience pool to compute and update the network parameters. The enhancement is realized by employing a maximum entropy model to learn mode-locked logic from the correlation between action outputs and the input states of the laser, the fastest mode-locked time is about 5.1 s and the average recovery time is 62.9 s. The empirical data in Fig. 12c(III) suggest that averaging a mere 37 trials in 80 randomized polarization state mode-locked tests. The system is capable of remote supervision, expands its application range, and can run in an unmanned environment. It is noteworthy that efficiency is contingent upon the constraints of the hardware platform, where leveraging FPGA for data acquisition and processing can precipitate a time reduction of at least 60%^221^.

The study of Sun et al. advanced a deep learning strategy that combines time-domain and spectral information to accurately search for soliton states within fiber lasers^13^. The approach generates the target pulse from white noise by controlling the temporal and spectral characteristics, avoiding the randomness of certain parameters that were encountered in prior research. Additionally, they have introduced the Phase Perceptron Algorithm (PPA), a novel numerical approach that deduces the phase distribution iteratively, offering in distinctive alternative to frequency-resolved optical gating and focusing specifically on retrieving the initial spectral phase distribution. This research contributes to a deeper understanding of the dynamics of mode-locked pulses, enabling precise control of pulse dynamics for applications in optical communication and nonlinear optics among others.

Pu et al. proposed a scheme for modeling femtosecond MLFLs using RNNs, with emphasis on the LSTM variant^234^. Their study enhances two key laser parameters (cavity length and small signal gain) and integrates them with waveform data as additional LSTM inputs, enabling the model to generalize over varying cavity lengths and small signal gains of the laser resonator. Additionally, the LSTM model infers 500 round-trip waveforms in under 0.1 s, achieving a computational speed ~146 times faster than the split-step Fourier method on a Central Processing Unit. This represents a substantial advancement in simulation efficiency, paving the way for rapid modeling and analysis of complex laser dynamics.

In the same year, they overcame the limitation of manual tuning that previously affected single-cavity dual-comb lasers by combining a real-time control module with memory-aided intelligent search, developing the first intelligent single-cavity dual-comb source capable of locating the desired state from CW state with an average time of 2.48 s^235^. The work significantly advances the technology for AML of dual-comb sources. Distinct from the aforementioned studies, Zhao et al. proposed a machine learning iterative optimization method based on Gaussian processes for the study of mode-locked formation in all-PM linear cavity fiber lasers^236^. The algorithm used in the experiment converged rapidly after only 30 runs and simulated mode-locked pulses generated from the noise that highly matched the output spectrum and pulse energy of the experimental results with the optimized parameters. Furthermore, they describe the intracavity dynamics under group velocity mismatch, proving that pulse trapping caused by cross-phase modulation leads to overcompensating timing synchronization between orthogonal polarization components.

Luo et al. pioneered the development of the shortest Praseodymium (Pr) laser to date, producing ~100 fs pulses by implementing a symmetric dispersion management scheme with soft role judging algorithm^237^. Their study marks a significant stride in the evolution of AML, showcasing the formidable potential of artificial intelligence in the regulation of ultrafast solid-state lasers. Integrating machine learning or deep learning with various models enables efficient learning and prediction, through training on large datasets, it rapidly learns the optimal parameters and conditions for mode-locking, facilitating the transition into a mode-locked state to output mode-locked pulses, achieving efficient prediction and optimization, handling complex nonlinear problems with strong adaptability and generalization capabilities. The trained model can achieve rapid real-time prediction and adjustment, making it suitable for dynamic mode-locking conditions.

Through a comprehensive analysis of the aforementioned references^221,232,233,238^ as presented in Table 2 that the implementation of various control techniques and algorithms facilitates the optimization of laser performance across different application scenarios, including enhancements in output power, reductions in pulse width, and increases in repetition frequency. The integration of intelligent algorithms proves particularly beneficial in achieving more stable and efficient laser operation, as these algorithms employ data-driven optimization strategies that efficiently navigate extensive polarization parameter spaces, with the speed of reaching a mode-locked state improving as the algorithms become more sophisticated, currently achieving millisecond-level responses (200 ms)^233^. Furthermore, the majority of current AML systems integrate the combination of EPC or optical slides and polarizers as essential components of AML technology, with EPCs possessing the capability to dynamically adjust the polarization state, thereby enabling precise control over the polarization direction and amplitude of electronic signals across multiple frequency ranges. Through algorithmic self-optimization of operational parameters, EPCs can achieve performance adaptability under varying environmental conditions, thereby enhancing the stability and reliability of mode-locking systems. When appropriately matched with the algorithms, diverse hardware integration methods foster a co-design approach between algorithms and hardware, which can potentially accelerate optimization processes and reduce energy consumption during mode-locking, thereby advancing the development of automated and energy-efficient ultrafast laser technologies. Pre-programming circuits to enable active fine-tuning helps counteract environmental fluctuations, such as temperature and vibration, correcting for variable sensitivities and environmental dependencies, ultimately leading to the creation of mode-locked lasers with enhanced robustness against environmental perturbations.

**Table 2:** Performances of AML technology combining different algorithms

Algorithms	Laser system	Control regimes	Repetition rate	Targeted parameters	Speed	Pulse width	Output power	Refs.
Traversal algorithm	NPE-based	LC installed on U-bench	8 MHz	FML	-	170 fs	2.5 mW	^98^
	NPE-based dispersion-mapped YDFL	EPC	-	NLP, ML,	a few min	-	178–235 mW	^208^
	NPR-based	MPC	81 MHz	ML	-	-	-	^205^
	all-fiber ring laser	EPC	-	ML	-	310 fs 250 fs	about 6 mW 2–5mW	^196^
	YDFL	EPC	61.6 MHz	ML	-	-	~20 mW	^56^
	EDFL	EPC	74.6 MHz	ML	-	-	4 mW	^203^
	NPR-based	EPC	62.5 MHz (ML)	QS, QML, ML	-	0.4 μs	∼3 mW	^204^
	NPR-based	EPC	6.238 MHz	Steady ML	-	-	-	^40^
EA/GA	NPE-based	EPC	-	QML, ML,	~30 min	150 ps	-	^212^
	NPE-based	EPC	25 kHz	QS	-	30 μs		^209^
	NOLM-based	EPC	7.4 MHz	QS, ML, NLP	~30 min	150 fs		^214^
	NPR-based EDFL	EPC	~6.17 MHz (FML) ~12.34 MHz (HML)	QS, FML, 2nd-order HML	1.01 s (FML), 28.21 s (2nd-order HML), 4.45 s (QS)	-	-	^210^
	NPR ANDi fiber laser	MPC	-	FML, single CW, double CW	-	-	-	^194^
	NPE-based TDFL	MPC	-	CW, QS, QML, ML, NLP	-	325 fs (Gaussian fitting)	57.7 mW (NLP)	^268^
	NPE-based TDFL	MPC		NLP	-	224 fs	-	^238^
	NPE-based	Waveplates, Polarizers	-	high-energy ML	simulation	-	-	^211^
GA and hill-climbing algorithm	NPE ANDi fiber oscillator	LCs	59.5 MHz	QS, ML	~30 s	90 fs	400–650 mW	^215^
HLA	NPR-based EDFL	EPC	7.2 MHz (FML) 115 kHz (QS) 200 kHz (QML)	FML, HML, QS, QML	3.1 s (mean of initial lock time of FML), 14.8 ms (recovery time)	-	-	^192^
DELAY algorithm	NPR-based UFL	EPC	39.46 MHz	Remote ML control, FML, QS,	5.8 s (average time) 1.948 s (average recovery time)	316 fs	-	^231^
MDRL	ANDi fiber laser	EPC	~13.5 MHz (FML) ~27 MHz (HML)	QS, QML, HML, FML	690 ms (average time) 200 ms (minimum)	14.2 ps (FML)	2 mW (prediction)	^233^
SAC-based algorithm	NPR-based EDFL	EPC	7.96 MHz	ML	5.1 s, 62.9 s (average recovery time)	-	-	^221^
DL-MPC	NPR-based	Polarizer, Waveplates	-	Stable ML	-	-	-	^199^
ESC algorithm	NPR-based	Waveplate, Polarizer	-	high-energy FML	Simulation	-	-	^223^
sparse search algorithm	Ring laser with saturable absorber	Waveplates, Polarizer	-	ML	Simulation	-	-	^217^

It is noteworthy that the construction of current AML systems predominantly employs fiber optic structures. As of 2024, a limited number of researchers have begun utilizing wavelength precision to classify speckle patterns, progressively applying various intelligent algorithms to identify different mode-locking states (such as QS, HML, and multi-pulse mode-locking) within solid-state lasers^239^, achieving fully automated output and stable mode-locking within 40 s. In contrast, the cavity structure of solid-state mode-locking lasers is relatively complex, with the alignment and relative positioning of different components significantly affecting the performance of the laser. Thus, aligning solid-state laser cavities requires considerable time and effort. Consequently, increasing research efforts in alternative structures has become a focal point for future investigations.

## Conclusion and discussion

In this review, we provide a comprehensive overview of the third-order nonlinear optics, the advances of mode-locking techniques, and AML. We have synthesized the fundamental principles of SA and RSA pivotal to current mode-locking techniques, observing that an increase in pump power and variations in material thickness can induce a reciprocal transformation between SA and RSA. Furthermore, we have detailed the evolution, merits, and drawbacks of various mode-locked methodologies, briefly delineated active mode-locking and hybrid mode-locking techniques while accentuated several configurations pertinent to PML and proffering solutions to their inherent challenges of susceptibility to environmental disturbance and integration complexities. AML addresses the challenges associated with MPC adjustments in traditional MLFL systems, such as time-consuming operations and instability due to environmental and stress factors, has yielded significant research outcomes.

Throughout the evolution of AML technology, algorithmic advancements have progressed from simple, inefficient exhaustive searches to sophisticated EA, machine learning and deep learning, reducing the search duration and recover time in vast, high-dimensional parameter spaces (14.8 ms)^192^. For hardware perspective, real-time control is employed due to their superior time efficiency. By incorporating more controllable variables like pump power, various feedback signals, more nuanced control can be enabled by AML over traditional MLFLs, paving the way for smarter systems. Additionally, The intricate interplay between nonlinearity, dispersion, gain and loss within the resonator gives rise to complex solitons dynamics, achieving continuous transmission of the soliton pulse in the laser^240^. Soliton molecules are an inherent structure in ultrafast lasers^241,242^, which contain abundant nonlinear dynamics, including rogue waves^243^, NLP^244–247^, soliton explosions^248–251^ and chaotic states^252^. In-depth study of soliton molecules is of great significance for understanding nonlinear dynamics between solitons and the intrinsic physical mechanism of ultrafast lasers^253,254^. The application of TS-DFT in AML offers novel insights into ultrafast internal transition dynamics, shedding light on mechanisms beyond the reach of conventional MLFLs. Consequently, this represents a fertile area of investigation, ripe for extensive research exploration.

While rapid advancements in reducing response times, inducing recovery time from loss of lock and identifying pulse states, intelligent laser researches currently have some shortcomings:

1. Intelligent laser researches are predominantly concentrated on NPR, with limited application of other mode-locking techniques.
2. Despite numerous intelligent optical control devices, system designs and algorithms have achieved seminal breakthroughs in simulations, yet empirical validation remains unattained.
3. The TS-DFT technique, a powerful analytical tool for various non-steady-state DS dynamics, its tunability is limited outside of polarization states, lacking adequate spectral control.
4. High-resolution and real-time considerations are paramount when configuring closed-loop feedback systems and hardware, especially for industrial lasers which often necessitate a singular mode-locked state, requiring rapid self-start and recovery and highly integrated design.
5. Limited by the existing electronic technology, the processing speed and memory capacity of the computing module cannot be improved indefinitely.
6. When the initial random polarization state is significantly far from the target state, substantial number of adjustment steps can be required, increasing the time to achieve mode-locked status and the restoration of mode-locked state.

Conducting essential in-depth analysis of the limitations in various technological solutions, we present the following rational prospects concerning the design and application of future intelligent lasers:

1. There remains potential for future integration of alternative mode-locking technologies to examine the varying performance parameters under different mode-locking mechanisms.
2. Researchers corroborate the viability of integrating algorithms through experimental trials.
3. Integrating high-speed oscilloscopes can allow real-time monitoring of complex nonlinear dynamics, facilitating the analysis and modulation of ultrafast pulses.
4. The design scheme of embedded chip and integrated printed circuit board effectively plays the advantage of embedded reduced instruction set efficiency and is widely used.
5. Integration of different mode-locking mechanisms with high-performance computing as computational modules to enhance the performance of system.
6. Further exploration is needed to develop superior algorithmic models to overcome the impact of the response time and precision of electronic components on systems that generate ultrafast pulse.
7. Prospective developments could incorporate a variety of parameters into feedback control mechanisms in the experiments, such as pump power, cavity length, etc., potentially achieving breakthroughs in recovery times, system stability, pulse width, etc.

The aforementioned considerations unequivocally indicate the necessity for continued research to refine AML technologies for diverse applications to offer pragmatic guidance for the design and application of future intelligent lasers, thereby fostering interdisciplinary exploration in optical science^255–258^, integrated strategies^259–261^, information and data processing^262–264^ and computer engineering domains^265–267^.
