# Author Correction: Advancements in ultrafast photonics: confluence of nonlinear optics and intelligent strategies

**DOI:** 10.1038/s41377-025-01868-0

**Published:** 2025-08-04

**Authors:** Qing Wu, Liuxing Peng, Zhihao Huang, Xiaolei Liu, Meng Luo, Danheng Gao, Haoran Meng

**Affiliations:** 1 Heilongjiang Province Key Laboratory of Laser Spectroscopy Technology and Application, Harbin University of Science and Technology, Harbin, 150080 China; 2 State Key Laboratory of Applied Optics, Changchun Institute of Optics, Fine Mechanics and Physics, Chinese Academy of Sciences, Changchun, 130033 China

**Keywords:** Ultrafast lasers, Mode-locked lasers

Correction to: *Light: Science & Applications*,

10.1038/s41377-024-01732-7, published online 25 February 2025

After publication of this article^1^, we noticed that the institutional affiliations of several co-authors were incorrect in the published paper. To ensure the accuracy, we would like to respectfully request a correction of the author affiliations as outlined below:

The affiliations of the following authors were inadvertently listed incorrectly: Meng Luo, Danheng Gao and Haoran Meng. The correct affiliation for Meng Luo, Danheng Gao and Haoran Meng should be: State Key Laboratory of Applied Optics, Changchun Institute of Optics, Fine Mechanics and Physics, Chinese Academy of Sciences, Changchun 130033, China.

Additionally, to avoid confusion and to ensure that all affiliations accurately reflect the authors’ current institutional associations, we have decided to remove the third affiliation (Wang Da-Heng Center of Quantum Control, Harbin University of Science and Technology, Harbin 150080, China) from the affiliation list. The institutional attribution for Qing Wu, Liuxing Peng, Zhihao Huang and Xiaolei Liu should be: Heilongjiang Province Key Laboratory of Laser Spectroscopy Technology and Application, Harbin University of Science and Technology, Harbin 150080, China.

Correspondingly, at “Author details”, it should be changed as:

^1^Heilongjiang Province Key Laboratory of Laser Spectroscopy Technology and Application, Harbin University of Science and Technology, Harbin 150080, China.

^2^State Key Laboratory of Applied Optics, Changchun Institute of Optics, Fine Mechanics and Physics, Chinese Academy of Sciences, Changchun 130033, China.

The original manuscript has been corrected.
